# Clinicopathological features and prognostic roles of KRAS, BRAF, PIK3CA and NRAS mutations in advanced gastric cancer

**DOI:** 10.1186/1756-0500-7-271

**Published:** 2014-04-29

**Authors:** Naoki Takahashi, Yasuhide Yamada, Hirokazu Taniguchi, Masaru Fukahori, Yusuke Sasaki, Hirokazu Shoji, Yoshitaka Honma, Satoru Iwasa, Atsuo Takashima, Ken Kato, Tetsuya Hamaguchi, Yasuhiro Shimada

**Affiliations:** 1 Gastrointestinal Oncology Division, National Cancer Center Hospital, 5-1-1 Tsukiji, Chuo-ku, Tokyo 104-0045, Japan; 2 Division of pathology, National Cancer Center Hospital, 5-1-1 Tsukiji, Chuo-ku, Tokyo 104-0045, Japan

## Abstract

**Background:**

RAS-RAF-MEK-ERK and PI3K-AKT pathways form a significant cascade for potential molecular target therapy in advanced cancer. The clinical significance of mutations in these genes in advanced gastric cancer (AGC) is uncertain.

**Methods:**

We collected formalin-fixed, paraffin-embedded and fresh frozen tumor samples from AGC patients and analyzed the *KRAS, NRAS, BRAF* and *PIK3CA* mutations by direct-sequencing. We retrospectively investigated the clinicopathological features of these mutations in AGC patients, and selected patients with metastatic gastric cancer.

**Results:**

Among 167 AGC patients, mutations of *KRAS* codons 12/13 (*N* = 8/164, 4.9%), *PIK3CA* (*N* = 9/163, 5.5%), and *NRAS* codon 12/13(*N* = 3/159, 1.9%) were detected. Comparison of the clinicopathological features of the mutated *KRAS, PIK3CA, NRAS* genes with an all-wild type of these genes showed that the frequency of the intestinal type was significantly higher in patients whose tumor tissue contained *KRAS* mutations (*P* = 0.014). Among 125 patients with metastatic gastric cancer, patients with *NRAS* codon 12/13 mutations in their tumors had shorter overall survival compared with *NRAS* wild-type patients (MST: 14.7 vs 8.8 months, *P* = 0.011). By multivariate analyses, *NRAS* codon 12/13 mutation was an indicator for poor prognosis in patients with metastatic gastric cancer (adjusted HR 5.607, 95% CI: 1.637-19.203).

**Conclusions:**

Our study indicated that mutations of *KRAS, PIK3CA* and *NRAS* were rare in AGC. *NRAS* mutations were likely to associate with poor prognosis in metastatic state of AGC patients, but further validation of other research is required.

## Background

Gastric cancer is the second leading cause of cancer death worldwide with approximately 989,600 new cases and 738,000 deaths per year, accounting for about 8 percent of new cancers [1]. The highest incidence rates are in Eastern Asia, the Andean regions of South America, and Eastern Europe, while the lowest rates are in North America, Northern Europe, and most countries in Africa and South Eastern Asia.

Owing to development of systemic chemotherapy, the survival time for advanced gastric cancer (AGC) has been improved during the past decade. A fluoropyrimidine and platinum regimen is a standard first-line chemotherapy in HER2-negative metastatic gastric cancer (mGC) patients, and trastuzumab added to XP is a standard chemotherapy in HER2-positive mGC patients in Japan [2-5]. Although some AGC patients obtained clinical benefit of systemic chemotherapy, most of the patients did not attain a clinically satisfactory outcome. Novel treatment of mGC with more effective and less toxic chemotherapy regimens was required.

Phase III trials of molecular therapy with mTOR inhibitor, anti-VEGF antibody, anti-EGFR antibodies were reported in AGC or gastro-esophageal cancer, but these drugs could not be demonstrated to have significant efficacy [6,7]. Recently, ramcirumab, anti-VEGFR target monoclonal antibody, was reported to improve the survival in chemotherapy-refractory mGC patients. It would be a significant therapeutic advantage to identify effective biomarkers in order to match the responsive cancer cells with the appropriate molecular target drug and elucidate further mechanisms associated with the resistance to chemotherapy.

The mitogen-activated protein kinase (MAPK) is part of a significant intracellular signal pathway that regulates diverse cellular functions including cell proliferation, cell cycle regulation, cell survival, angiogenesis, and cell migration [8]. The Ras proteins were initially identified as the transforming components of oncogenic viruses, whereas *NRAS* was identified as the transforming component of a neuroblastoma. Ras mutations are found in up to 30% of all cancers and are particularly common in pancreatic and colon cancers. Raf is recruited to the cell membrane through binding to Ras and is activated in a complex process involving phosphorylation and multiple cofactors. *BRAF* mutations have a narrow distribution, but are prevalent in a few specific malignancies such as melanoma, papillary thyroid cancer, and low-grade ovarian cancer [9-11]. The importance of phosphoinositide 3-kinase (PI3Ks) in cancer was confirmed by the discovery that the *PIK3CA* gene, encoding the PI3K catalytic subunit p110α, is frequently mutated in some of the most common human tumors [12]. These genetic alterations of *PIK3CA* consist exclusively of somatic missense mutations clustered in two “hotspot” regions in exons 9 and 20, corresponding to the helical and kinase domains of p110α, respectively [13].

Recently, the use of *KRAS*, *BRAF*, *PIK3CA* and *NRAS* as biomarkers for molecular target therapy in solid tumors has been widely discussed. Several small-scale biomarker analyses of *KRAS*, *BRAF* and *PIK3CA* mutations were reported previously in AGC [14-16]. The clinical significance of these mutations in AGC patients is not already clarified, and further investigations of these intracellular molecular changes are required.

In the present study, we conducted a genomic analysis of *KRAS*, *BRAF*, *PIK3CA* and *NRAS* mutations in order to investigate the clinicopathological features and prognostic role of gene mutations in AGC patients.

## Methods

### Patients and sample collection

**Figure 1:**
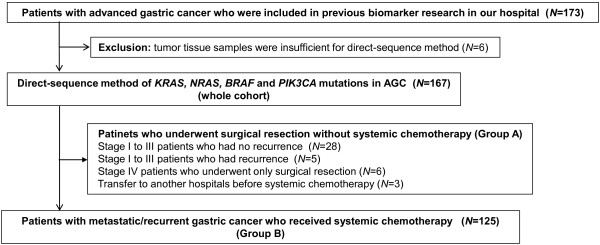
Diagram of this study.

All the data were extracted from the database of our department, and chart review was done for each patient in order to obtain important information. We collected tissue samples for analysis of the gene mutation status of *KRAS, BRAF, PIK3CA* and *NRAS*. Tissue samples consisted of samples used in previous biomarker research in our institution [2,17] and of fresh frozen tissue samples, which were obtained from previous surgical resections of AGC in our institution. Tumor tissue samples of 173 AGC patients were gathered, but insufficient samples from 6 patients were excluded. Finally, we used 167 tissue samples from AGC patients and investigated gene mutations of *KRAS*, *BRAF*, *PIK3CA* and *NRAS* by the direct-sequencing method (whole cohort ). Among 167 AGC patients, 42 patients underwent surgical resection without systemic chemotherapy (non-metastatic cohort as group A) and 125 patients with metastatic gastric cancer received systemic chemotherapy (metastatic cohort as group B). A diagram of the present study is shown in Figure 1. Informed consent of using patient’s tumor tissues was confirmed from all of the patients who participated in the study, which was conducted with the approval of the Institutional Ethical Review Board of the National Cancer Center in accordance with the Helsinki declaration of 1975 (as revised in 1983).

### Genomic analyses of KRAS, BRAF, PIK3CA and NRAS

DNA samples were extracted from formalin-fixed, paraffin-embedded (FFPE) and fresh frozen tumor tissue sections. Tumor cell-rich areas in the hematoxylin and eosin section were marked under a microscope, and tissue was scratched from the area of another deparaffinized unstained section. DNA from pieces of the scratched tissue sample was isolated using the QIAamp DNA FFPE Tissue Kit (QIAGEN KK, Tokyo, Japan). Exon 2 (codon 12, 13), exon 3 (codon 61), exon 4 (codon 146) of *KRAS*gene and exon 15 (codon 600) of *BRAF* gene and exon 9 (codon 542, 545), exon 20 (codon 1047) of *PIK3CA*gene and exon 2 (codon 12, 13), exon 3 (codon 61) of *NRAS* gene were amplified by PCR (the GeneAmp PCR System 9700 thermal cycler). The PCR products were visualized using agarose gel electrophoresis with ethidium bromide staining and directly sequenced using an ABI 3130x/ Genetic Analyzer (Life Technologies Japan (Applied Biosystems), Tokyo, Japan) according to the manufacturer’s instructions.

### Treatment

A total of 125 patients in group B received systemic chemotherapy. Regimens of first-line chemotherapy consisted of CP (n = 42, 33.6%), S-1 (n = 39, 31.2%), 5-FU (n = 10, 24.0%), a combination of 5-FU and methotrexate (n = 10, 8.0%), Paclitaxel (n = 3, 2.4%) and XP and/or bevacizumab (n = 1, 0.8%). Key anti-cancer drugs for AGC in Japan are fluoropyrimidine (5-FU, S-1, capecitabine), cisplatin, irinotecan and taxane. During the whole course of systemic chemotherapy in group B, the proportions of patients receiving 5-FU, cisplatin, irinotecan and taxane were 85.6% (n = 107/125), 52.8% (n = 66/125), 60.8% (n = 76/125), 36.0% (n = 45/125), respectively. In addition, 22 patients (17.6%) received all key anti-cancer drugs, 37 patients (29.6%) received 3 of the key drugs, 30 patients (24.0%) received 2 of the key drugs, and 36 patients (28.8%) received only one of the key anti-cancer drugs. The schedules and doses of CP, S-1 and 5-FU were based on the previous reports [17]. Paclitaxel as monotherapy was repeated three times weekly for 4 weeks and the docetaxel as monotherapy was given by intravenous infusion once every 3 weeks.

### Statistical analyses

We evaluated the proportion of each *KRAS*, *BRAF*, *PIK3CA* and *NRAS* mutation in whole cohort and the prognostic values of these mutations, which were adjusted variables of patients’ characteristics in terms of overall survival (OS) in metastatic group B. OS was defined as the interval from initiation of first-line chemotherapy to death or last follow up.

We performed statistical analyses by SPSS statistical software, version 19 (IBM, Tokyo, Japan). Differences in the distribution of variables were evaluated using the Fisher exact test or chi-square test, as appropriate. Median survival time (MST) was estimated by the Kaplan–Meier method and survival curves were compared by the log-rank test. All tests were two-sided and a p-value <0.05 was defined as statistically significant. We estimated hazard ratio (HR) and the corresponding 95% confidence interval (CI) for OS using univariate and multivariate analyses by the Cox proportion hazard models. Variables in this analysis included age (≥median/<median), gender (male/female), ECOG PS (0/1-2), histological type of Lauren classification (intestinal type/diffuse type), number of metastatic sites (single/multiple).

## Results

Direct sequencing of tissue samples in group B determined the proportion of each of *KRAS*, *BRAF*, *PIK3CA* and *NRAS* (Table 1). Mutations of *KRAS* codon 12 (3.7%, n = 6/164) and *KRAS* codon 13 (1.2%, n = 2/164), *PIK3CA* exon 9 (4.9%, n = 8/163), *PIK3CA* exon 20 (0.6%, n = 1/163) and *NRAS* mutations (1.9%, n = 3/159) were detected. On the other hand, mutations in *KRAS* codon 61, *KRAS* codon 146, *BRAF* V600E, and *NRAS* codon 146 were not detected. *KRAS* codon 12 mutations consisted of G12D (35G > A, n = 4), G12S (34G > A; n = 1) and G12N (34 35GG > AA; n = 1), and codon 13 mutations consisted of G13S (37G > A; n = 1) and G13V (38 39GC > TT; n = 1). *PIK3CA* exon 9 mutations consisted of E542K (1624G > A; n = 2), E545K (1633G > A; n = 5), and E545D (1635G > C; n = 1), and exon 20 mutations consisted of H1047R (3140A > G; n = 1). *NRAS* mutations consisted of G12S (34G > A; n = 1) and G13S (37G > A; n = 1). There were 70 patients whose tumor tissue contained all-wild types of *KRAS* (exon2, 3, 4), *BRAF* (exon 15), *PIK3CA* (exon9, 20) and *NRAS* (exon2, 3). Among the AGC patients whose tumor tissue contained gene mutations, multiple mutations of *KRAS* codon 13, *PIK3CA* codon 545 and *NRAS* codon 12 were detected in only one case.

**Table 1:** **Results of gene mutations of *KRAS, BRAF, PIK3CA *** **and *NRAS *** **in AGC patients**

**Gene mutations**	**Proportion (%)**	**Insufficient DNA samples**
***KRAS *** **codon 12**	6/164 (3.7)	3/167
***KRAS *** **codon 13**	2/164 (1.2)	3/167
***KRAS *** **codon 61**	0/131 (0.0)	36/167
***KRAS *** **codon 146**	0/137 (0.0)	30/167
***BRAF *** **V600E**	0/136 (0.0)	31/167
***PIK3CA *** **exon 9**	8/163 (4.9)	4/167
***PIK3CA *** **exon 20**	1/163 (0.6)	4/167
***NRAS *** **codon 12**	1/159 (0.6)	8/167
***NRAS *** **codon 13**	2/159 (1.3)	8/167
***NRAS *** **codon 61**	0/135 (0.0)	32/167

The clinicopathological features of each gene mutation compared with the all-wild type are summarized in Table 2. The median ages of patients whose tumor tissue contained mutations of *KRAS*, *PIK3CA* and *NRAS* (54.5, 58.0 and 56.0 years, respectively) were lower than that of patients containing all-wild types of *KRAS, BRAF, PIK3CA* and *NRAS* (median age, 64.0 years)*.* There was no significant difference, except for histological type, among variables of clinicopathological features such as gender, age, ECOG PS and the numbers of metastatic sites. Compared with all-wild type patients, the proportions of intestinal type were higher in patients with *KRAS* codon 12/13 mutation (p = 0.014). The histological tumor type in all patients whose tumor tissue contained *NRAS* mutations was the diffuse type of adenocarcinoma.

**Table 2:** Comparison of clinocopathological features by gene mutations status compared with all-wild type in patients with AGC patients

	**All wild-type**	***KRAS *** **codon 12/13**		***PIK3CA *** **exon 9/20**		***NRAS *** **codon12/13**	
	***KRAS, BRAF, NRAS, PIK3CA***	**Mutant type**	***P*-value**	**Mutant type**	***P *–value**	**Mutant type**	***P*-value**
**Number of patients**	70	8		9		3	
**Median age**	64.0	54.5		58.0		56.0	
**Gender (%)**							
Male	49 (70.0)	7 (87.5)	0.429	8 (88.9)	0.432	2 (87.5)	1.000
Female	21 (30.0)	1 (12.5)		1 (11.1)		1 (12.5)	
**ECOG PS (%)**							
0	38 (54.3)	3 (37.5)	0.466	4 (44.4)	0.727	1 (33.3)	0.476
1≦	32 (45.7)	5 (62.5)		5 (55.6)		2 (66.7)	
**Histological type (%)**							
Intestinal type	20 (28.6)	6 (75.0)	0.014	4 (44.4)	0.443	0 (0.0)	0.556
Diffuse type	50 (71.4)	2 (25.0)		5 (55.6)		3 (100.0)	
**No. of metastatic site (%)**							
1	54 (77.1)	8 (100.0)	0.195	8 (88.9)	0.675	3 (100.0)	1.000
2≦	16 (22.9)	0 (0.0)		1 (11.1)		0 (0.0)	
**Metastatic lesion (%)**							
Lymph node	41 (58.6)	3 (37.5)	0.348	6 (66.7)	0.717	2 (66.7)	0.851
Liver	14 (20.0)	3 (37.5)		3 (33.3)		1 (33.3)	
Lung	2 (2.9)	1 (12.5)		0 (0.0)		0 (0.0)	
Peritoneal dissemination	18 (25.7)	1 (12.5)		1 (11.1)		0 (0.0)	
Other	3 (4.3)	0 (0.0)		0 (0.0)		0 (0.0)	

*Abbreviations*: *ECOG PS* Eastern Cooperative Oncology Group performance status. We described the clinical data of 90 patients, and we excluded the patients whose tumor tissues had no gene mutations but at least one gene could not be evaluated.

The background characteristics of metastatic gastric cancer patients are shown in Table 3. Most patients (98.4%) were ECOG PS 0/1, and only 2 patients (1.6%) were ECOG PS 2. A total of 71 patients (60.0%) had the histologically diffuse tumor type, and 50 patients (40.0%) had the intestinal type of adenocarcinoma. As for the number of metastatic sites, 30 patients (24.0%) had metastasis to multiple organs, and 95 patients (76.0%) had metastasis to one organ. Common metastatic sites were lymph nodes, peritoneum and liver.

**Table 3:** Background characteristics of patients with total cohort, group A and group B

	**Total**	**Group A (non-metastatic group)**	**Group B (metastatic group)**
**Number of patients**	167	42	125
**Age (median)**	64.0	65.0	63.0
**Gender (%)**			
Male	124 (74.3)	29 (69.0)	95 (76.0)
Female	43 (15.7)	13 (31.0)	30 (24.0)
**ECOG PS (%)**			
0	79 (47.3)	33 (78.6)	46 (36.8)
1	86 (51.5)	9 (21.4)	77 (61.6)
2	2 (1.2)	0 (0.0)	2 (1.6)
**Histological type (%)**			
Intestinal type	60 (35.9)	10 (23.8)	50 (40.0)
Diffuse type	107 (64.1)	36 (76.2)	71 (60.0)
**T-stage**			
T1	9 (5.4)	7 (16.7)	2 (1.6)
T2	34 (20.4)	9 (21.4)	25 (20.0)
T3	101 (60.5)	22 (52.4)	79 (63.2)
T4	23 (13.8)	4 (9.5)	19 (15.2)
**N-stage**			
Nx	4 (2.4)	2 (4.8)	2 (1.6)
N0	22 (13.2)	12 (28.6)	10 (8.0)
N1	56 (33.5)	16 (38.1)	40 (32.0)
N2	53 (31.7)	9 (21.4)	44 (35.2)
N3	32 (19.2)	3 (19.2)	29 (23.2)
**Metastatic lesion (%)**			
≦1	135* (80.8)	40* (95.2)	95 (76.0)
2≦	32 (19.2)	2 (4.8)	30 (24.0)
**Metastatic site (%)**			
Lymph node	92* (55.1)	19* (45.2)	73 (58.4)
Liver	34 (20.3)	2** (4.8)	32 (25.6)
Lung	8 (4.8)	0 (0.0)	8 (6.4)
Peritoneal dissemination	43 (25.7)	3** (7.1)	40 (32.0)
Other	6 (3.6)	0 (0.0)	6 (4.8)

*Abbreviations*: *ECOG PS* Eastern Cooperative Oncology Group performance status. Staging (TNM classification) is diagnosed by Japanese Classification of Gastric Carcinoma (The 13th Edition) , *including metastasis to regional lymph nodes. **resectable lesion.

The MST in metastatic GC patients was14.1 months (95% CI: 12.5-15.7 months). Patients whose tumor tissue contained a *NRAS* codon 12/13 mutation had a significantly shorter OS compared with those carrying the *NRAS* wild type (8.8 month vs. 14.7 months, p = 0.011, log-rank test). On the other hand, there was no significant difference in OS between patients with wild type or mutant type of *KRAS* codon 12/13 (13.2 vs. 15.2 months, p = 0.775) and *PIK3CA* exon 9/20 (13.6 vs. 9.4 months, p = 0.286).

We evaluated the prognostic factors for OS by univariate and multivariate analyses in metastatic group B. There was no significant difference among variables of patient background characteristics, but patients with ECOG PS 1/2 (HR: 1.380, 95% CI: 0.941-2.024) and multiple metastatic sites (HR: 1.452, 95% CI: 0.956-2.206) had a tendency to have shorter OS by univariate analyses. By multivariate analysis, 2 or more metastatic sites (HR: 1.613, 95% CI: 1.047-2.484) was an independent variable in prediction of shorter OS.

HRs and 95% CIs of variables of gene mutations (*KRAS* codon 12/13, *PIK3CA* exon 9/20 and *NRAS* codon 12/13) were adjusted by age, gender, ECOG PS, histological type and metastatic sites. Among these mutations, the *NRAS* codon 12/13 mutation was an independent value in prediction of shorter OS by multivariate analysis (adjusted HR: 5.607, 95% CI: 1.637-19.203).

## Discussion and conclusions

Our analysis suggested that mutations of *KRAS* codon 12/13 and *PIK3CA* exon 9/20 (codons 542, 545 and 1047) were not observed frequently in AGC patients, and *BRAF* mutations (V600E) were not detected. To our knowledge, clinicopathological features and prognostic roles of *KRAS* codon 61, *KRAS* codon 146, *NRAS* codon 12/13 and *NRAS* codon 61 have not been reported in AGC patients previously. Mutations in *KRAS* codon 61, *KRAS* codon 146 and *NRAS* codon 61 were not detected, but *NRAS* codon 12/13 mutation was detected in 3 of 159 patients (1.9%) in the present study. Interestingly, the intestinal type of adenocarcinoma was found more frequently in patients whose tumor tissue contained *KRAS* codon 12/13 mutations and diffuse type of adenocarcinoma was observed in all 3 patients whose tumor tissue contained *NRAS* codon 12/13 mutations. In addition, *NRAS* mutations were likely to be associated with shorter OS in metastatic GC patients. Oncogenic mutations often point to the presence of a therapeutic target that might be amenable to directed therapeutic intervention. Molecular target therapy of MARK and PI3K-Akt cascades is an attractive strategy in AGC patients.

In advanced gastro-esophageal adenocarcinoma, the frequency of *KRAS* codon 12/13 mutations was approximately 3.4 to 9.4% according to biomarker analyses of small-size clinical trials of anti-EGFR antibodies treatment [18-20]. Our study indicated that *KRAS* mutations were observed in 4.9% of AGC patients, which is similar to the results of these clinical trials. Several retrospective analyses have reported on frequencies and clinicopathological features of *KRAS* mutations in gastric cancer [14-16]. According to these reports, the most common mutation of *KRAS* codon 12 was G12D, and all mutations of *KRAS* codon 13 were G13D. Our study also indicated that G12D mutations were the most common mutations, and we found in 4 of 6 tumor tissue samples containing the *KRAS* codon 12 mutations. On the other hand, the G13D *KRAS* mutation was not detected in our study (G13V and G13S), unlike observations in previous reports. In these previous reports, most of the tumor tissues containing the *KRAS* codon 12/13 were of the intestinal histological type. Zhao W et al. suggested that there were significant differences in the presence of *KRAS* mutations according to tumor site (antrum vs. non-antrum, p = 0.002) and status of microsatellite instability (MSI) (MSI-high vs. MSI-loss, p = 0.000076). The frequency of the intestinal type of adenocarcinoma was significantly higher than that of the diffuse type of adenocarcinoma in our study. There was no definite evidence for a role of *KRAS* mutations in prediction and prognosis of success of molecular target therapy in AGC. Recently, the randomized, multicenter, phase II/III REAL-3 trial, which tested the addition of panitumumab to a modified epirubicin, oxaliplatin, and capecitabine (EOC) regimen, was reported, and a multivariate biomarker analysis of 200 patients indicated that *KRAS* mutation was a prognostic factor for OS [7]. In a large-scale clinical trial of treatment of gastric-esophageal cancer with anti-EGFR antibodies, *KRAS* mutations also appeared to have significant prognostic value, but we need to verify this result by further biomarker analyses of the treatment of molecular therapy in AGC.

Gene amplifications, deletions and more recently, somatic missense mutations in the *PIK3CA* gene have been reported in several malignancies, including cancers of the colon, breast, lung, brain, liver and stomach [21,22]. In gastric cancer, previous reports indicated that the frequency of *PIK3CA* mutations (exons 9 and 20) was 2.5 to 10.6% [12,14,20]. Nine of 168 AGC patients (4.5%) had *PIK3CA* mutations in our study, and there was no great difference compared with previous reports. Some previous reports suggested a better prognosis for breast cancer patients with *PIK3CA* mutations, whereas others suggested that *PIK3CA* mutations were associated with a worse prognosis in colorectal cancer, endometrial cancer and lung cancer [23-26]. Multivariate analyses of the REAL-3 trial indicated that *PIK3CA* mutations indicated poor OS prognosis in the treatment with anti-EGFR antibodies in gastro-esophageal cancer. Our data suggested that *PIK3CA* mutation was not associated with the prognosis in mGC patients treated with systemic chemotherapy, although this study was not a large-scale analysis. Clinical trials of molecular therapy that targets PI3K-AKT-mTOR pathways have been initiated recently, thus results of biomarker analyses of these pathways are required.

*NRAS* mutations have been mainly described in melanoma and leukemia [27,28], but the prognostic significance in these malignancies has been unclear. Some previous reports have suggested an association between *NRAS* mutations and a poor prognosis in melanoma and a poor response to anti-EGFR antibodies in colorectal cancer [29]. Our study indicated that the frequency of *NRAS* mutations (codons 12 and 13) was 1.9% in AGC and was lower than that seen in other malignancies. Interestingly, multivariate analyses showed that small groups of *NRAS* mutations had poor prognosis in metastatic gastric cancer patients who received systemic chemotherapy in present study. We must consider a probable bias of small sample size of *NRAS* mutations. On the other hand, patient’s characteristics of *NRAS* mutations were younger and smaller number of metastasis site than all wild-type patients. There was no significant difference in chemotherapeutic regimens and number of key drugs between patients with *NRAS* mutations and all-wild type patients. Previously, *NRAS* mutations have not been investigated routinely as a prognostic biomarker in clinical trials of AGC. In addition to having prognostic significance, that *NRAS* mutations as well as *KRAS, BRAF* and *PIK3CA* mutations would be better to be discussed as potential target for molecular therapy in AGC patients.

The present study has several limitations. First, the chemotherapeutic regimens in our study were previous standard regimens in the mid-2000s in Japan. Second, we could not conclude definitely from the data in our study alone that *NRAS* mutations have prognostic significance because of the low frequencies of *NRAS* mutations and the large confidence intervals. Third, there were some insufficient samples and we needed to unify the better methods of sample’s preservation.

In conclusions, our study indicated that the frequencies of gene mutations of *KRAS, BRAF* and *PIK3CA* were very similar to those observed in previous reports. *NRAS* mutations were rare in AGC patients, but may have a prognostic value in mGC patients who receive systemic chemotherapy. We hope that our results will contribute to future molecular therapy of AGC patients.

## Abbreviations

AGC: Advanced gastric cancer; mGC: Metastaric gastric cancer; EGFR: Epidermal growth factor receptor; FFPE: Formalin-fixed, paraffin-embedded; ECOG PS: Eastern Cooperative Oncology Group performance status; PFS: Progression free survival; OS: Overall survival; HR: Hazard rate; CI: Confidence interval; MST: Median survival time; EOC: Pirubicin, oxaliplatin, and capecitabine.

## Competing interests

The authors declare that they have no competing interests.

## Authors’ contributions

NT contributed to the drafting of this manuscript and data collection, and NT, YY contributed to the study design and statistical analysis. HT, MF, YS, HS, YH, SI, AT, KK, TH, SS contributed to analysis of the data and interpretation of the findings. All authors have read and approved of the submission of the final manuscript.
